# Triglyceride-Glucose Index and New-Onset Atrial Fibrillation in ST-Segment Elevation Myocardial Infarction Patients After Percutaneous Coronary Intervention

**DOI:** 10.3389/fcvm.2022.838761

**Published:** 2022-03-08

**Authors:** Yang Ling, Cong Fu, Qun Fan, Jichun Liu, Ling Jiang, Shengxing Tang

**Affiliations:** Department of Cardiology, Yijishan Hospital, Wannan Medical College, Wuhu, China

**Keywords:** TyG index, new-onset atrial fibrillation, ST-segment elevation myocardial infarction, percutaneous coronary intervention, insulin resistance, prognosis

## Abstract

**Background:**

New-onset atrial fibrillation (NOAF) is associated with worse prognostic outcomes in cases diagnosed with ST-segment elevation myocardial infarction (STEMI) patients after percutaneous coronary intervention (PCI). The triglyceride-glucose (TyG) index, as a credible and convenient marker of insulin resistance, has been shown to be predictive of outcomes for STEMI patients following revascularization. The association between TyG index and NOAF among STEMI patients following PCI, however, has not been established to date.

**Objective:**

To assess the utility of the TyG index as a predictor of NOAF incidence in STEMI patients following PCI, and to assess the relationship between NOAF and long-term all-cause mortality.

**Methods:**

This retrospective cohort research enrolled 549 STEMI patients that had undergone PCI, with these patients being clustered into the NOAF group and sinus rhythm (SR) group. The predictive relevance of TyG index was evaluated through logistic regression analyses and the receiver operating characteristic (ROC) curve. Kaplan-Meier curve was employed to explore differences in the long-term all-cause mortality between the NOAF and SR group.

**Results:**

NOAF occurred in 7.7% of the enrolled STEMI patients after PCI. After multivariate logistic regression analysis, the TyG index was found to be an independent predictor of NOAF [odds ratio (OR): 8.884, 95% confidence interval (CI): 1.570–50.265, *P* = 0.014], with ROC curve analyses further supporting the predictive value of this parameter, which exhibited an area under ROC curve of 0.758 (95% CI: 0.720–0.793, *P* < 0.001). All-cause mortality rates were greater for patients in the NOAF group in comparison with the SR group over a median 35-month follow-up period (log-rank *P* = 0.002).

**Conclusions:**

The TyG index exhibits values as an independent predictor of NOAF during hospitalization, which indicated a poorer prognosis after a relatively long-term follow-up.

## Introduction

One of the most frequent complications that affects an estimated 5–11% of ST-segment elevation myocardial infarction (STEMI) cases following percutaneous coronary intervention (PCI) is new-onset atrial fibrillation (NOAF) (1, 2). NOAF incidence in this context has been linked to higher rates of morbidity, mortality and prolonged hospitalization, which are largely attributable to the hemodynamic instability and pro-thromboembolic effects (3–7). The precise etiological basis of NOAF in STEMI patients following PCI is complex and influenced by a range of risk factors such as aging, female sex, hypertension and heart failure (5, 8, 9). Reliable clinical predictors of NOAF are, however, lacking at present. There is thus a clear need to identify tools for the detection of STEMI patients who are at an elevated risk of NOAF incidence following PCI in order to better guide their care.

Insulin resistance (IR), as a metabolic disorder in which the normal utilization of glucose by the body is disrupted, resulting in altered lipid processing and reduced glycogen synthesis, has been closely linked to the onset of cardiovascular disease (CVD) (10). A recent work further suggested that even after adjustment for potential confounding factors there is a significant correlation between IR and atrial fibrillation (AF) incidence in non-diabetic Asian populations (11). As IR has the potential to contribute to atrial remodeling, that may explain this regulatory relationship and the observed rise in AF susceptibility (12).

A hyperinsulinemic-euglycemic clamp remains the benchmark test for IR at present, but it is a complex and dangerous procedure that is rarely implemented in the clinic (13). In addition, the homeostasis model assessment of IR (HOMA-IR) approach is frequently employed to gauge patient IR status, but its use in large-scale epidemiological research is limited by the need to measure insulin levels in fasting blood samples (14, 15). In an effort to develop a more reliable and robust alternative means of detecting IR, the triglyceride-glucose (TyG) index was developed based on a combination of measurements of fasting plasma glucose (FPG) and triglyceride (TG). The TyG index has been shown to be well-correlated with HOMA-IR and hyperinsulinemic-euglycemic clamp results and is consequently a promising indicator of IR status (16–18). Prior research also suggested that TyG index values are independent predictors of an elevated risk of major adverse cardiac and cerebrovascular events in STEMI patients following PCI (19). The specific utility of the TyG index as a predictor of NOAF incidence in this patient population, however, has yet to be explored. The current exploration was therefore developed to investigate the closeness between the TyG index and NOAF occurrence among STEMI patients that have undergone PCI.

## Materials and Methods

**Figure 1:**
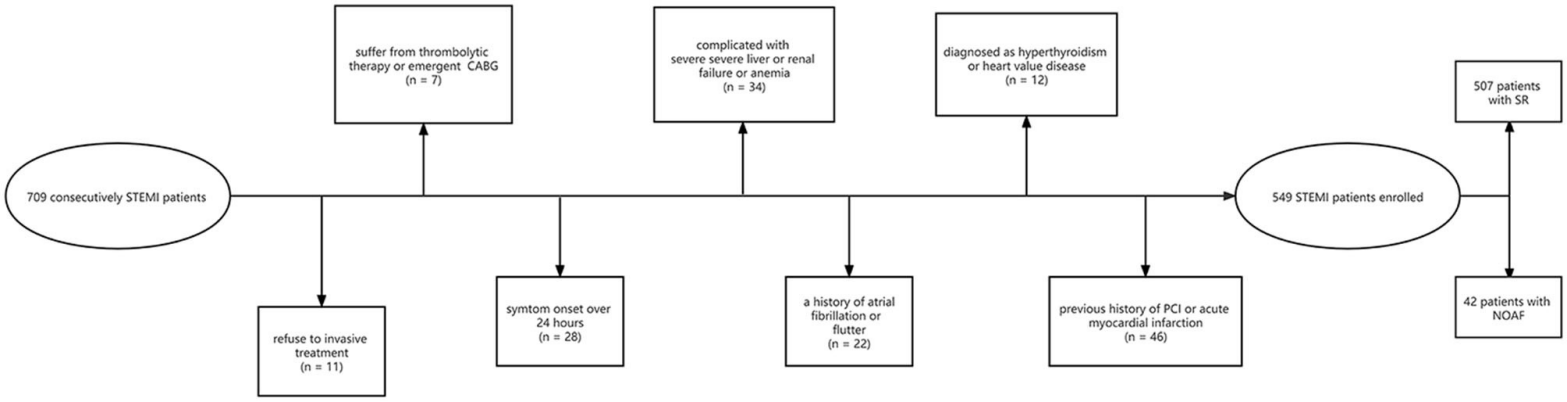
Flow chart of patients. STEMI, ST-segment elevation myocardial infarction; CABG, coronary artery bypass grafting surgery; PCI, percutaneous coronary intervention; SR, sinus rhythm; NOAF, new-onset atrial fibrillation.

For the present study, 709 consecutive STEMI patients > 18 years old who had been admitted to the Yijishan hospital department of cardiology between February 2016 and February 2020 were retrospectively enrolled. Patients were diagnosed with STEMI as per the European Society of Cardiology standard (20). Patients were excluded from this analysis if: (1) they refused to undergo invasive treatment, (2) they had been admitted over 24 h after the onset of symptoms, (3) they underwent thrombolytic therapy or emergent coronary artery bypass grafting surgery (CABG), (4) they exhibited STEMI complicated by severe liver or renal failure or anemia, (5) they demonstrated background of AF or atrial flutter, (6) they exhibited hyperthyroidism or heart valve disease defined as valvular regurgitation or stenosis, or (7) they had any history of PCI or acute myocardial infarction (AMI). In sum, 549 patients were qualified to participate in the present research bases on these criteria (Figure 1). None of the patients had received lipid-lowering drugs before. The ethics committee of the Yijishan hospital approved this study, which was consistent with the Declaration of Helsinki. Due to the retrospective nature of these analyses, the requirement for informed satisfaction was waived.

Data regarding patient demographics, clinical data, electrocardiogram (ECG) results, laboratory parameters, angiographic and echocardiography findings, duration of hospitalization, and in-hospital outcomes were obtained from electronic medical records. Blood routine examination, such as white blood cell (WBC), neutrophil, hemoglobin, platelet, was measured with the Mindray BC-5380 hematology analyzer using the manufacturer recommended reagents. An automated biochemistry analyzer (Roche Cobas 801, Germany) was used to assess TG and FPG values in overnight fasting blood samples collected from the cubital vein of each patient within 12–24 h post-symptom onset. TyG index values were evaluated as follows: ln [fasting TG (mg/dL) × FPG (mg/dL)/2] (16). The estimated glomerular filtration rate (eGFR) was calculated by the Modification of Diet in Renal Disease (MDRD) equation (21). The transthoracic echocardiography was performed using a commercially available iE33 instruments (Philips Medical Systems, Koninklijke, Netherlands) for each patient within 24 h following admission. All patients underwent coronary angiography and PCI via the radial approach. The characteristics of coronary stenosis [the culprit vessel, Thrombolysis In Myocardial Infarction (TIMI) grade flow, and The SYNergy between Percutaneous Coronary Intervention with TAXus and cardiac surgery (SYNTAX) score] and the length of stents were evaluated by two expert interventional cardiologists blinded to patient clinical information (22).

The diagnosis of AF was complied with the consensus guidelines (23). Telemetry ECG was used to monitor all patients while they remained hospitalized in the cardiac care unit following PCI, with a standard 12-lead ECG being performed once per day until patients were discharged or when any new symptoms were observed. NOAF was explained as the detection of AF lasting ≥30 s during post-PCI hospitalization that subsequently spontaneously revered to sinus rhythm or responded to antiarrhythmic drug-mediated cardioversion.

Analyzed patient outcomes included in-hospital mortality, cardiogenic shock, pulmonary edema, post-PCI ventricular tachycardia, intra-aortic ballon pump (IABP) implantation, stroke and long-term all-cause mortality. Patients' follow-up outcomes were collected from patient medical records or telephone-based interviews. June 16, 2021 was the end time point of follow-up.

The Kolmogorov-Smirnov assessment was employed to evaluate whether data were normally distributed. Those obtained results that were normally distributed were given as means ± standard deviation (SD), while they were otherwise reported as medians with interquartile ranges. Continuous data were scrutinized via Student's *t*-tests or Mann-Whitney *U*-tests, while categorical variables were compared via Fisher's exact analysis or chi-squared tests. Multivariate logistic regression analysis was performed incorporating significant variables from univariate analyses (*P* < 0.1) in an effort to identify independent predictors of NOAF. The curve of receiver operating characteristic (ROC) was implemented to gauge the value of the TyG index as a predictor of NOAF incidence. Youden's index was used to select an appropriate TyG index cut-off value. All-cause mortality rates were compared between patients with and without NOAF using Kaplan-Meier curves. SPSS 23.0 (IBM) was used to analyze all data, with *P* < 0.05 as the threshold of significance.

## Results

In sum, 549 contributors were enrolled in this research and separated into NOAF and sinus rhythm (SR) groups, with NOAF patients accounting for 7.7% (42/549) of the overall cohort. Patient baseline characteristics are compiled in Table 1. Relative to cases in the SR group, those in the NOAF group tended to be older, had a higher body mass index (BMI), increased diabetes mellitus (DM) incidence, and more often exhibited a Killip class ≥ II. There were no discrepancies between these patient groups in relation to gender, hypertension, or history of smoking or alcohol intake, or the medications usage including angiotensin-converting enzyme inhibitor or angiotensin receptor blocker (ACEI/ARB), beta blocker, mineralocorticoid receptor antagonist (MRA) or statin.

**Table 1:** Baseline characteristics of patients between SR and NOAF group.

**Variables**	**SR (*n* = 507)**	**NOAF (*n* = 42)**	***P***
Age (years)	63.0 (53.0–72.0)	69.5 (65.8–75.2)	<0.001
Female (*n*%)	99.0 (19.5)	12.0 (28.6)	0.161
Hypertension (*n*%)	243.0 (47.9)	26.0 (61.9)	0.082
Diabetes mellitus (*n*%)	129.0 (25.4)	25.0 (59.5)	<0.001
Killip class ≥ II	145.0 (28.6)	23.0 (54.8)	<0.001
Smoking (*n*%)	266.0 (52.5)	20.0 (47.6)	0.546
Alcohol intake (*n*%)	248.0 (48.9)	22.0 (52.4)	0.666
Body mass index (Kg/m^2^)	25.1 (24.1–26.4)	25.5 (24.7–27.2)	0.027
**Biochemical markers**			
Hemoglobin (g/l)	141.0 (129.0–154.0)	134.5 (121.8–144.3)	0.041
White blood cell (10^9^/l)	10.8 (8.8–12.7)	13.2 (10.5–16.2)	<0.001
Neutrophil (10^9^/l)	8.6 (6.7–10.7)	11.2 (8.2–13.7)	<0.001
Platelet (10^9^/l)	176.0 (140.0–220.0)	151.0 (133.3–198.0)	0.040
Glucose (mmol/l)	5.52 (4.76–6.86)	7.87 (5.63–10.89)	<0.001
Total cholesterol (mmol/l)	4.13 (3.54–4.84)	3.95 (3.28–4.55)	0.111
Triglyceride (mmol/l)	1.33 (0.98–2.01)	2.09 (1.51–2.67)	<0.001
HDL-c (mmol/l)	1.16 (1.02–1.32)	1.26 (1.07–1.41)	0.042
LDL-c (mmol/l)	2.36 (1.97–2.89)	2.05 (1.72–2.55)	0.007
TyG index	8.73 (8.33–9.19)	9.38 (9.03–10.00)	<0.001
eGFR (ml/min*1.73 m^2^)	121.23 (97.46–147.87)	95.31 (74.38–132.03)	0.002
Peak CK (*10^3^)	1.55 (0.87–2.85)	1.57 (0.61–2.64)	0.566
Uric acid (μmol/l)	353.7 (287.8–430.3)	361.8 (305.6–428.8)	0.644
Albumin (g/l)	36.70 ± 3.83	35.80 ± 4.34	0.149
**Coronary angiography**			
TIMI flow grade <3 pre-PCI	395.0 (77.9%)	36.0 (85.7%)	0.237
Stent length (mm)	29.0 (21.0–33.0)	29.0 (23.0–36.0)	0.052
SYNTAX score	19.5 (14.0–23.5)	20.0 (15.4–25.5)	0.134
Culprit vessels			0.405
LAD (*n*%)	277.0 (54.6)	20.0 (47.6)	
LCX (*n*%)	39.0 (7.7)	2.0 (4.8)	
RCA (*n*%)	191.0 (37.7)	20.0 (47.6)	
**Echocardiography**			
Left atrium diameter (mm)	36.0 (33.0–40.0)	39.5 (37.0–42.0)	<0.001
LVEF (%)	51.0 (47.0–56.0)	48.0 (44.3–55.0)	0.012
**In-hospital outcomes**			
Hospitalization days	12.0 (11.0–14.0)	16.0 (11.0–18.2)	0.001
Stroke (*n*%)	5.0 (1.0)	2.0 (4.8)	0.094
Pulmonary edema (*n*%)	51.0 (10.1)	18.0 (42.9)	<0.001
Cardiogenic shock (*n*%)	57.0 (11.2)	14.0 (33.3)	<0.001
Death (*n*%)	15.0 (3.0)	6.0 (14.3)	0.001
Post-PCI VT (*n*%)	12.0 (2.4)	6.0 (14.3)	<0.001
IABP implantation (*n*%)	5.0 (1.0)	3.0 (7.1)	0.011
**Medications use at discharge**			
ACEI/ARB (*n*%)	404.0 (79.7)	34.0 (81.0)	0.844
Beta blockers (*n*%)	362.0 (71.4)	29.0 (69.0)	0.746
MRA (*n*%)	218.0 (43.0)	19.0 (45.2)	0.778
Statin (*n*%)	499.0 (98.4)	40.0 (95.2)	0.378

*SR, sinus rhythm; NOAF, new-onset atrial fibrillation; HDL-c, high-density lipoprotein cholesterol; LDL-c, low-density lipoprotein cholesterol; TyG index, Triglyceride-glucose index; eGFR, estimated glomerular filtration rate; CK, creatine kinase; TIMI, Thrombolysis In Myocardial Infarction; SYNTAX, SYNergy between Percutaneous Coronary Intervention with TAXus and cardiac surgery; LAD, left anterior descending coronary artery; LCX, left circumflex coronary artery; RCA, right coronary artery; LVEF, left ventricular ejection fraction; PCI, percutaneous coronary intervention; IABP, intra-aortic ballon pump; ACEI, angiotensin-converting enzyme inhibitor; ARB, angiotensin receptor blocker; MRA, mineralocorticoid receptor antagonist*.

Substantial discrepancies were observed between the NOAF and SR groups in relation to white blood cell (WBC), neutrophil, hemoglobin, platelet, glucose, triglyceride, high-density lipoprotein cholesterol (HDL-c), low-density lipoprotein cholesterol (LDL-c), eGFR, and TyG index values, while albumin, uric acid, peak creatine kinase (CK) and total cholesterol levels did not differ between these groups. Specifically, WBC, neutrophil, glucose, triglyceride, and TyG index were higher in NOAF patients relative to SR patients, whereas platelet and hemoglobin levels, and the values of eGFR were decreased in individuals exhibiting NOAF. Coronary angiography and echocardiography analyses revealed that patients in the NOAF group exhibited worse cardiac function and a larger left atrial diameter. No differences in SYNTAX scores, stent length, culprit vessels, or TIMI flow grade <3 prior to PCI were detected when comparing the NOAF and SR groups (Table 1).

NOAF patients exhibited significantly increased rates of in-hospital mortality, cardiogenic shock, pulmonary edema, IABP implantation, and post-PCI ventricular tachycardia as compared to SR patients, in addition to exhibiting a longer average duration of hospitalization (Table 1).

In univariate logistic regression analyses, age, hypertension, DM, a Killip class ≥II, BMI, hemoglobin, WBC, neutrophil, platelet, glucose, total cholesterol, triglyceride, TyG index, HDL-c, LDL-c, eGFR, stent length, SYNTAX score, left atrium diameter, and left ventricular ejection fraction values were all identified as predictors of NOAF incidence among STEMI cases following PCI. Of these, a multivariate regression analysis revealed age, TyG index, and eGFR levels to be independent predictors of NOAF incidence in this patient population (Table 2).

**Table 2:** Logistic analysis for predictors of NOAF in STEMI patients following PCI.

**Variables**	**OR**	**95% CI**	***P***
**Univariate analysis**			
Age	1.056	1.026–1.087	<0.001
Gender	0.607	0.300–1.227	0.164
Hypertension	1.765	0.925–3.371	0.085
Diabetes mellitus	4.309	2.255–8.236	<0.001
Killip class ≥ II	3.022	1.598–5.717	0.001
Smoking	0.824	0.439–1.547	0.546
Alcohol intake	1.149	0.612–2.157	0.666
Body mass index	1.221	1.015–1.470	0.034
Hemoglobin	0.984	0.965–1.003	0.094
White blood cell	1.176	1.088–1.270	<0.001
Neutrophil	1.127	1.049–1.211	0.001
Platelet	0.995	0.988–1.001	0.083
Glucose	1.177	1.093–1.268	<0.001
Total cholesterol	0.747	0.538–1.037	0.081
Triglyceride	1.478	1.205–1.813	<0.001
HDL-c	2.979	0.953–9.315	0.060
LDL-c	0.536	0.334–0.859	0.009
TyG index	3.551	2.314–5.449	<0.001
eGFR	0.988	0.979–0.997	0.009
Peak CK	1.000	1.000–1.000	0.964
Uric acid	1.002	0.999–1.004	0.122
Albumin	0.941	0.867–1.002	0.149
TIMI flow grade <3 pre-PCI	0.588	0.242–1.430	0.242
Culprit vessel LAD^a^	0.710	0.160–3.157	0.653
Culprit vessel LCX^a^	1.450	0.760–2.769	0.260
Stent length	1.023	1.004–1.043	0.018
SYNTAX score	1.046	1.000–1.094	0.052
Left atrium diameter	1.132	1.061–1.209	<0.001
LVEF	0.943	0.904–0.984	0.007
ACEI/ARB	1.084	0.487–2.411	0.844
Beta blocker	0.894	0.452–1.767	0.746
MRA	1.095	0.582–2.061	0.778
Statin	0.321	0.066–1.561	0.159
**Multivariate analysis** ^**b**^			
Age	1.058	1.013–1.105	0.011
TyG index	8.884	1.570–50.265	0.014
eGFR	0.987	0.976–0.998	0.022

*NOAF, new-onset atrial fibrillation; STEMI, ST-segment elevation myocardial infarction; PCI, percutaneous coronary intervention; OR, odds ratio; CI, confidence interval; HDL-c, high-density lipoprotein cholesterol; LDL-c, low-density lipoprotein cholesterol; TyG index, Triglyceride-glucose index; eGFR, estimated glomerular filtration rate; CK, creatine kinase; TIMI, Thrombolysis In Myocardial Infarction; SYNTAX, SYNergy between percutaneous coronary intervention with TAXus and cardiac surgery; LVEF, left ventricular ejection fraction, ACEI, angiotensin-converting enzyme inhibitor; ARB, angiotensin receptor blocker; MRA, mineralocorticoid receptor antagonist*.

a *Compared with culprit vessel RCA*.

b *Age, Hypertension, Diabetes mellitus, Body mass index, Hemoglobin, White blood cell, Neutrophil, Platelet, Glucose, Triglyceride, Total cholesterol, HDL-c, LDL-c, eGFR, TyG index, Left atrium diameter, LVEF, SS, Stent length and Killip class ≥ II had been included in the multivariate logistic regression analysis*.

**Figure 2:**
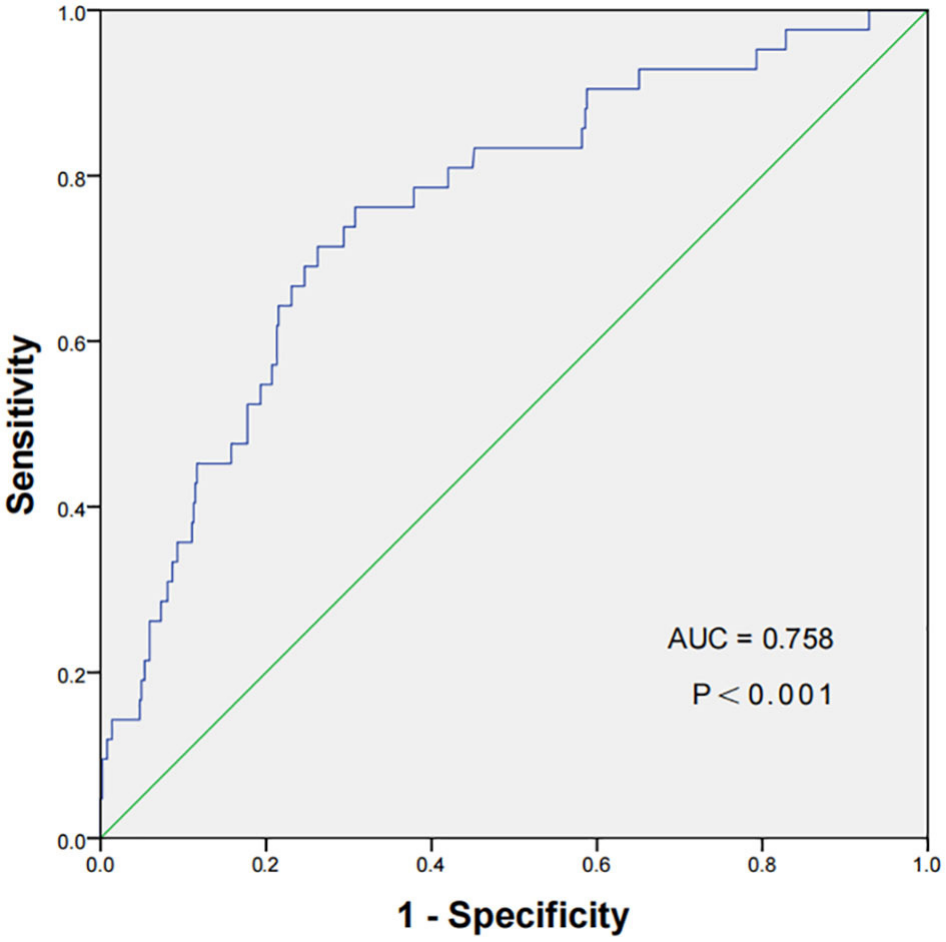
The ROC curve of the TyG index to predict NOAF in STEMI patients following PCI. The cut-off value of TyG index was 9.15, with a sensitivity of 71.43% and a specificity of 73.77% (area under the receiver curve 0.758, 95% CI: 0.720–0.793, *P* < 0.001). ROC, receiver operating characteristic; TyG, triglyceride-glucose Index; NOAF, new-onset atrial fibrillation; STEMI, ST-segment elevation myocardial infarction; PCI, percutaneous coronary intervention.

A ROC curve of the TyG index for the prediction of NOAF in STEMI patients following PCI had been displayed in Figure 2, which revealed that the cut-off value of TyG index was 9.15, with a sensitivity of 71.43% and a specificity of 73.77%, respectively. In addition, the area under the receiver curve of TyG index for predicting the occurrence of NOAF in STEMI patients after PCI was 0.758 [95% confidence interval (CI): 0.720–0.793, *P* < 0.001].

Next, patients were separated into cohorts with low or high TyG index values based on a TyG index cut-off value derived from an ROC analysis. On average, cases with a high TyG index were younger, more likely to be female, had higher DM incidence rates, and exhibited higher WBC, neutrophil, hemoglobin, glucose, total cholesterol, triglyceride, and albumin levels and left atrial diameter values as compared to cases with a low TyG index value. NOAF incidence and in-hospital mortality rates were also greater among cases with a high TyG index (Supplementary Table 1).

**Figure 3:**
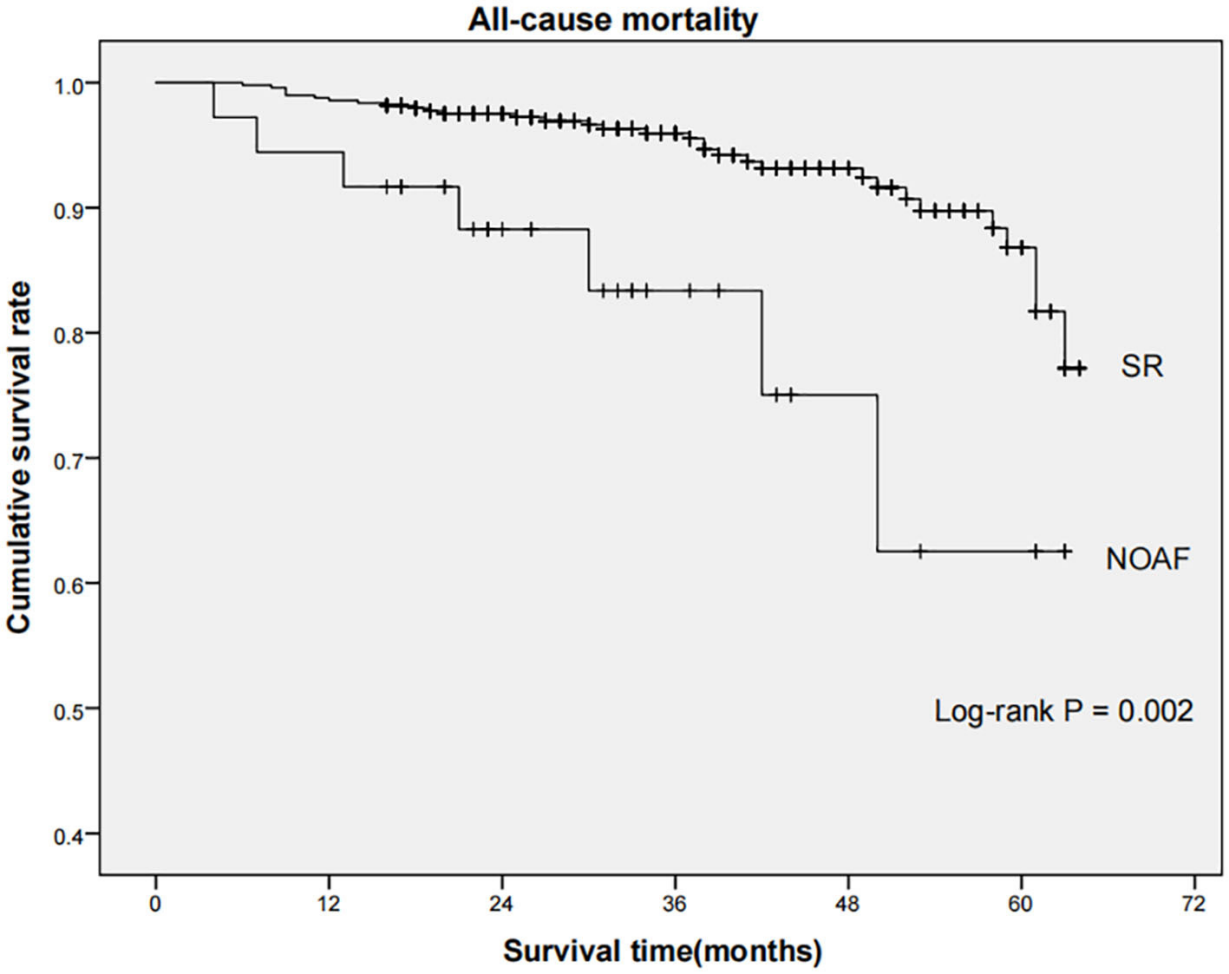
Comparison of all-cause mortality between SR and NOAF group. Kaplan-Meier curves indicated that the rate of all-cause mortality among patients in NOAF group was significantly higher than that in SR group (log-rank *P* = 0.002). SR, sinus rhythm; NOAF, new-onset atrial fibrillation.

Long-term outcome data were available for 528 patients, while 21 patients experienced in-hospital mortality. Over a median 35-month follow-up period, the rate of all-cause mortality in the NOAF and SR group was 19.4 and 6.5%, respectively, with the rate being substantially greater in the NOAF group (log-rank *P* = 0.002, Figure 3).

## Discussion

Herein, the TyG index was utilized as a surrogate biomarker for IR status in an effort to clarify the utility of IR as a predictor of NOAF in STEMI patients after PCI. Overall, these analyses confirmed that the TyG index offers value as an independent risk factor associated with post-PCI NOAF occurrence among STEMI patients, in addition to revealing that long-term all-cause mortality among NOAF patients were higher than those for SR patients. This study is the first to our knowledge to have demonstrated the value of the TyG index as a tool for gauging the risk of NOAF incidence among STEMI patients that have undergone PCI.

NOAF is a relatively common outcome observed after PCI in STEMI patients. We found that 7.7% of the STEMI patients in our study cohort developed NOAF during hospitalization, consistent with previously reported rates of AF development for similar patient populations (1, 2). Prior reports have demonstrated new-onset AF after STEMI revascularization is correlated with higher incidence of morbidity, mortality as well as prolonged hospital stay, consistent with the relevance of NOAF as a risk factor associated with greater clinical severity and a worse prognosis (4, 6, 7, 24, 25). Consistently, we found that patients in the NOAF group in our study cohort exhibited a longer duration of hospitalization and higher rates of both in-hospital mortality and long-term all-cause mortality as compared to SR patients. Therefore, identifying STEMI patients at elevated risk of NOAF after PCI is of clear clinical value as a means of ensuring inpatients receive appropriate care. Several variables have already been proposed to be associated with NOAF risk, including gender, advanced age, hypertension, DM, and congestive heart failure (5, 8, 26–28).

Recent researches explain that the TyG index is positively associated with metabolic risk factors and cardiovascular outcomes in different patient populations. For example, an analysis of 5,014 cases in the Vascular Metabolic CUN cohort revealed that elevated values of TyG index had been correlated with higher odds of CVD onset over a 10-year follow-up period (29). Zhao et al. further showed that higher TyG index was closely related to an elevated risk of nephric microvascular damage in elderly participants in the Northern Shanghai Study after controlling for potential confounding variables (30). Moreover, Chen et al. conducted a longitudinal survey of 7,428 individuals without DM over a median 3.4-year follow-up period and found TyG index to be positively correlated with a higher risk of new-onset DM among middle-aged and older adults in China (31). Jin et al. analyzed follow-up data over a 36-month period from 1,282 consecutive DM patients diagnosed with stable coronary artery disease, and found TyG index to be predictive of cardiovascular outcomes after adjusting for confounding risk factors (32). Luo et al. conducted a retrospective analysis of 1,092 STEMI patients over 1-year post-PCI follow-up period, and determined that higher TyG index values were predictive of higher rates of adverse clinical events (19). Another recent retrospective cohort study of 409 cases exhibiting hypertrophic obstructive cardiomyopathy following septal myectomy proposed the TyG index to be an independent predictor of postoperative NOAF (14). No prior studies, however, have directly assessed the relationship between TyG index and NOAF occurrence in STEMI patients following PCI. We herein found the levels of TyG index among NOAF patients in the present study cohort to be higher than those observed among SR patients. Consistently, patients with high TyG index were more likely to experience NOAF as compared to patients with low TyG index. Notably, we determined that a higher TyG index value was an independent risk factor for the incidence of NOAF after adjusting for possible confounding variables.

The TyG index is a composite metric that incorporates TG and FDP in an effort to provide an easily measured indicator of patient IR status. While the hyperinsulinemic-euglycemic clamp approach remains the benchmark means of assessing IR, it entails significant risk and complexity that render it unsuitable for routine clinical use (13). The HOMA-IR scale is an alternative approach to gauging patient insulin sensitivity, but necessitates measurements of fasting insulin levels which are not routinely conducted in department of cardiology, thus limiting its application in the evaluation of STEMI patients (15). The TyG index has emerged as a more convenient alternative approach to assessing patient IR status because it correlates well with both HOMA-IR and hyperinsulinemic-euglycemic clamp results and only requires routine measurements of TG and FPG values (16–18). The TyG index is thus a readily accessible biochemical marker of IR that is well-suited to routine clinical use.

The exact mechanistic basis for the correlation between the TyG index and AF development remains to be fully elucidated. Given that the TyG index serves as a surrogate for IR, this may underlie its association with NOAF in the present patient cohort. IR is a complex metabolic disorder in which tissues and organs exhibit impaired glucose uptake and/or utilization together with abnormal lipolysis owing to dysfunctional insulin signaling, ultimately resulting in compensatory hyperinsulinemia (10, 33). IR has been reported to be associated with increased inflammation, oxidative stress, cardiac hypertrophy and fibrosis, and the hyperphosphorylation of proteins associated with calcium handling, all of which have the potential to influence atrial electrical and structural remodeling, thus resulting in AF onset and maintenance (12, 34–36). Hyperinsulinemia has also been proposed to participate in the activation of sympathetic nervous and renin-angiotensin-aldosterone system, thereby contributing to atrial neural remodeling and a consequent rise in susceptibility to AF (37, 38). Maria et al. presented that IR can suppress atrial glucose transporter expression, contributing to the establishment of metabolic conditions conducive to AF occurrence (39). Despite this growing body of research regarding the relationship between IR and AF, however, further research will be essential to fully explore how the TyG index is linked to AF development.

There are multiple important clinical implications to these results. First, the TyG index is a convenient, easy to measure biochemical indicator that is ideally suited to use in routine clinical practice, thus enabling the efficient identification of patients at a high risk of NOAF incidence. By determining which patients are most likely to suffer from this adverse outcome, it may be possible to provide them with better individualized care such as prolonged ECG monitoring during hospitalization. Moreover, our results have the potential to expand current understanding regarding the pathological mechanisms underlying NOAF, thus suggesting potential avenues for the inhibition of this condition.

Several Limitations need to be taken into consideration in present study. For one, this was a single-center study focused solely on Chinese participants, thus limiting the degree to which these results can be generalized. Moreover, this was a retrospective analysis with a limited sample size, thus might limit a sound statistical analysis. All risk factors with the potential to influence IR, including hypertension or diabetes medications, dietary habits and lifestyle characteristics, were not assessed. The result was inherently susceptible to selection bias. Future large-scale prospective research is thus critical to validate and expand our result. In addition, the lack of fasting insulin levels and HOMA-IR may be another limitation. Lastly, TyG index values were only assessed at the time of hospitalization, and whether TyG index values during follow-up offer valuable insight into IR status remains to be established.

In summary, the TyG index represents an independent predictor of NOAF during hospitalization. Moreover, the patients with NOAF during hospitalization had worse prognosis after discharge.

## Data Availability Statement

The original contributions presented in the study are included in the article/Supplementary Material, further inquiries can be directed to the corresponding author/s.

## Ethics Statement

The studies involving human participants were reviewed and approved by Ethics Committee of Yijishan Hospital, Wannan Medical College. Written informed consent for participation was not required for this study in accordance with the national legislation and the institutional requirements.

## Author Contributions

YL designed the subject and wrote the manuscript. QF, JL, and LJ collected the data for the article. CF and ST analyzed the data and reviewed the manuscript. All authors contributed to the article and approved the submitted version.

## Conflict of Interest

The authors declare that the research was conducted in the absence of any commercial or financial relationships that could be construed as a potential conflict of interest.

## Publisher's Note

All claims expressed in this article are solely those of the authors and do not necessarily represent those of their affiliated organizations, or those of the publisher, the editors and the reviewers. Any product that may be evaluated in this article, or claim that may be made by its manufacturer, is not guaranteed or endorsed by the publisher.
