# Supplementary material for: Triglyceride-Glucose Index and New-Onset Atrial Fibrillation in ST-Segment Elevation Myocardial Infarction Patients After Percutaneous Coronary Intervention
Source: Front Cardiovasc Med. 2022 Mar 8;9:838761. doi: 10.3389/fcvm.2022.838761 (PMC8957253; doi:10.3389/fcvm.2022.838761)
Supplement: Supplementary file 1 [file Table_1.docx]

***Supplementary Table 1*.** Baseline characteristics of patients between low and high TyG index group.

| **Variables** | **Low TyG index group**  **(n = 386)** | **High TyG index group**  **(n = 163)** | P |
| --- | --- | --- | --- |
| Age (years) | 65.0 (55.0-73.0) | 58.0 (49.0-70.0) | 0.001 |
| Female (n%) | 66 (17.1) | 45 (27.6) | 0.005 |
| Hypertension (n%) | 182 (47.2) | 87 (53.4) | 0.183 |
| Diabetes mellitus (n%) | 54 (14) | 100 (61.3) | ＜ 0.001 |
| Killip class ≥ II | 119 (30.8) | 49 (30.1) | 0.858 |
| Smoking (n%) | 214 (55.4) | 72 (44.2) | 0.016 |
| Alcohol intake (n%) | 195 (50.5) | 75 (46.0) | 0.335 |
| Body mass index (Kg/m^2^) | 25.1 (24,1-26.4) | 25.4 (24.4-26.5) | 0.154 |
| **Biochemical markers** |  |  |  |
| Hemoglobin (g/l) | 140.0 (127.8-152.0) | 143.0 (129.0-157.0) | 0.039 |
| White blood cell (10^9^/l) | 10.7 (8.7-12.5) | 11.5 (9.6-14.1) | ＜ 0.001 |
| Neutrophil (10^9^/l) | 8.4 (6.8-10.5) | 9.7 (7.0-12.0) | 0.004 |
| Platelet (10^9^/l) | 172.0 (136.0-219.0) | 179.0 (146.0-215.0) | 0.302 |
| Glucose (mmol/l) | 5.21 (4.66-6.03) | 7.93 (6.00-11.61) | ＜ 0.001 |
| Total cholesterol (mmol/l) | 3.99 (3.38-4.69) | 4.43 (3.77-5.06) | ＜ 0.001 |
| Triglyceride (mmol/l) | 1.18 (0.91-1.53) | 2.55 (1.88-3.31) | ＜ 0.001 |
| HDL-c (mmol/l) | 1.18 (1.02-1.33) | 1.16 (1.04-1.34) | 0.981 |
| LDL-c (mmol/l) | 2.33 (1.90-2.87) | 2.39 (1.97-2.80) | 0.515 |
| TyG index | 8.56 (8.21-8.83) | 9.60 (9.33-10.00) | ＜ 0.001 |
| eGFR (ml/min*1.73m^2^) | 121.23 (98.37-149.67) | 113.77 (90.03-142.21) | 0.085 |
| Peak CK (*10^3^) | 1.55 (0.82-2.91) | 1.54 (0.96-2.71) | 0.628 |
| Uric acid (μmol/l) | 353.4 (292.7-421.8) | 355.6 (279.7-445.5) | 0.743 |
| Albumin (g/l) | 36.23 ± 3.91 | 37.59 ± 3.65 | ＜ 0.001 |
| **Coronary angiography** |  |  |  |
| TIMI flow grade ＜ 3 pre-PCI | 92 (23.8) | 26 (16.0) | 0.04 |
| Stent length (mm) | 29.0 (21.0-33.0) | 29.0 (23.0-33.0) | 0.475 |
| SYNTAX score | 19.5 (13.0-23.5) | 20.0 (15.0-23.5) | 0.195 |
| Culprit vessels |  |  | 0.159 |
| LAD (n%) | 216 (56.0) | 81 (49.7) |  |
| LCX (n%) | 24 (6.2) | 17 (10.4) |  |
| RCA (n%) | 146 (37.8) | 65 (39.9) |  |
| **Echocardiography** |  |  |  |
| Left atrium diameter (mm) | 36.0 (33.0-39.0) | 38.0 (34.0-40.0) | 0.002 |
| LVEF (%) | 51.0 (47.0-56.0) | 51.0 (45.0-55.0) | 0.568 |
| **In-hospital outcomes** |  |  |  |
| Hospitalization days | 12.0 (11.0-14.0) | 13.0 (10.0-15.0) | 0.563 |
| NOAF (n%) | 12.0 (3.1) | 29.0 (17.8) | ＜ 0.001 |
| Stroke (n%) | 5.0 (1.3) | 2.0 (1.2) | 0.948 |
| Pulmonary edema (n%) | 43.0 (11.1) | 26.0 (16.0) | 0.120 |
| Cardiogenic shock (n%) | 43.0 (11.1) | 28.0 (17.2) | 0.054 |
| Death (n%) | 10.0 (2.6) | 11.0 (6.7) | 0.020 |
| Post-PCI VT (n%) | 9.0 (2.3) | 9.0 (5.5) | 0.055 |
| IABP implantation (n%) | 3.0 (0.8) | 5.0 (3.1) | 0.053 |
| **Medications use at discharge** |  |  |  |
| ACEI/ARB (n%) | 303.0 (78.5) | 135.0 (82.8) | 0.249 |
| Beta blockers (n%) | 270.0 (69.9) | 121.0 (74.2) | 0.311 |
| MRA (n%) | 175.0 (45.3) | 62.0 (38.0) | 0.115 |
| Statin (n%) | 381.0 (98.7) | 158.0 (96.9) | 0.156 |

NOAF, new-onset atrial fibrillation; TyG index, Triglyceride-glucose index; TIMI, Thrombolysis In Myocardial Infarction; SYNTAX, SYNergy between Percutaneous Coronary Intervention with TAXus and cardiac surgery; LAD, left anterior descending coronary artery; LCX, left circumflex coronary artery; RCA, right coronary artery; LVEF, left ventricular ejection fraction; PCI, Percutaneous Coronary Intervention; IABP, intra-aortic ballon pump; ACEI, angiotensin-converting enzyme inhibitor; ARB, angiotensin receptor blocker; MRA, mineralocorticoid receptor antagonist.
